# Establishment of a Bacterial Infection Model Using the European Honeybee, *Apis mellifera* L

**DOI:** 10.1371/journal.pone.0089917

**Published:** 2014-02-24

**Authors:** Kenichi Ishii, Hiroshi Hamamoto, Kazuhisa Sekimizu

**Affiliations:** Laboratory of Microbiology, Graduate School of Pharmaceutical Sciences, University of Tokyo, Bunkyo-ku, Tokyo, Japan; National Institutes of Health, United States of America

## Abstract

Injection of human pathogenic bacteria (*Pseudomonas aeruginosa*, *Serratia marcescens*, *Salmonella enterica*, *Staphylococcus aureus*, and *Listeria monocytogenes*) into the hemocoel of honeybee (*Apis mellifera* L.) workers kills the infected bees. The bee-killing effects of the pathogens were affected by temperature, and the LD_50_ values at 37°C were more than 100-fold lower than those at 15°C. Gene-disrupted *S. aureus* mutants of virulence genes such as *agrA*, *saeS*, *arlR*, *srtA*, *hla*, and *hlb* had attenuated bee-killing ability. Nurse bees were less susceptible than foragers and drones to *S. aureus* infection. Injection of antibiotics clinically used for humans had therapeutic effects against *S. aureus* infections of bees, and the ED_50_ values of these antibiotics were comparable with those determined in mammalian models. Moreover, the effectiveness of orally administered antibiotics was consistent between honeybees and mammals. These findings suggest that the honeybee could be a useful model for assessing the pathogenesis of human-infecting bacteria and the effectiveness of antibiotics.

## Introduction

Bacterial infections represent one of the greatest threats to human health, and overcoming infectious diseases is a clinically important issue. The recent emergence of multi-drug resistant pathogens further increases the mortality and morbidity of bacterial infection [1]. Therapeutic effects of antibiotics against bacterial infections are defined not only by the antimicrobial mechanisms but also by the pharmacokinetics of the drugs in the host body [2]. Thus, in addition to ordinary *in vitro* screenings for antimicrobial substances, evaluations of therapeutic effects using whole animals is essential for the discovery of novel antibiotics that will benefit infected human patients. For *in vivo* assessment of candidate chemicals, mammals such as mice and rats have long been used as model animals. Over the last several years, however, infection experiments using a large number of mammals have been difficult to perform due to the high costs involved and the ethical issues regarding animal welfare [3]. Therefore, overcoming these problems by establishing novel lower animal models of *in vivo* bacterial infections has become an important goal.

Insects have many advantages as an infection model, such as low cost, few ethical issues, and ease of handling in pharmaceutical and infection experiments. We previously demonstrated that silkworms, *Bombyx mori*, are killed by injection of human pathogens into the bloodstream [4]–[9]. The silkworm infection model is useful for assessing the contribution of virulence factors, such as the two-component system regulators [10]–[14] and cytotoxins [12], to bacterial pathogenesis [5], [15]. We identified novel virulence genes conserved among several pathogenic bacteria using the silkworm as a host animal for screening [5]. Moreover, silkworms infected with bacteria are efficiently cured by administration of the same antibiotics used clinically to treat human patients [16], [17]. The amounts of antibiotics per animal weight required to prevent death (ED_50_) are comparable between infected silkworms and mammals [16], [17]. Moreover, basic mechanisms of the innate immune systems are conserved between vertebrates and insects [18], and we have identified a novel innate immune pathway involving the activation of an insect cytokine paralytic peptide in silkworms [19]–[21]. Thus, we consider that insects, including silkworms, are useful as alternative animal models for examining the therapeutic potential of drug candidates against human infectious microbes.

The honeybee, *A. mellifera* L., is a social insect that lives in a colony comprising individuals with varying tasks. In particular, worker honeybees shift their labors from nursing the blood by secreting royal jelly to guarding the hives and then to foraging for nectar and pollen according to their age after eclosion (*i.e.,* the emergence of an adult insect from the pupal case) [22]. Workers exhibit grooming, removal of mites from the body surfaces, and cleaning dead bees and feces inside the nests, which are important tasks for maintaining the hygienic environment [23]. On the other hand, although honeybees possess a battery of innate immune pathways similar to those found in *B. mori* and *Drosophila melanogaster*, the number of immune-related genes in the honeybee genome is lower than that in other insects [24]. Thus, the development of hygienic behaviors seems to be evolutionarily important for honeybees given the limited repertoire of immune factors within each individual [24]. Although silkworms and flies have been established as infection models, there are no previous reports describing the use of honeybees for quantitative assessment of pathogenesis of human-infecting pathogens and the effects of antibiotics. Taking advantage of well-established rearing methods of bees that enable us to easily obtain large numbers of animals, here we describe the establishment of an experimental system of bacterial infection and antibiotic treatment using the honeybee.

## Materials and Methods

### Insects, Bacteria, and Reagents

European honeybees, *Apis mellifera* L., were purchased from a local distributer (Kumagaya Honeybee Farm, Saitama, Japan) and maintained at the University of Tokyo as previously reported [25]. Beehives were placed outside at a temperature ranging from 25∼35°C in summer and 0∼10°C in winter. Nurse bees, foragers, and drones were selected as previously described [26].

*Escherichia coli* strain KP7600, *Pseudomonas aeruginosa* strain PAO1, *Salmonella enterica* serovar Typhimurium strain ATCC 14028s, *Staphylococcus aureus* strain Newman, and *Listeria monocytogenes* strain 104035 were grown in LB10 medium at 37°C for 12 to 18 h. *Serratia marcescens* strain 2170 was grown in LB10 medium at 30°C for 12 to 18 h. *S. aureus* disruption mutants of parent strain NCTC8325-4 were constructed as previously described [15]. Bacterial cultures were centrifuged and the cells were suspended in saline (0.9% NaCl).

Vancomycin hydrochloride, gentamycin sulfate, and kanamycin sulfate were purchased from Wako and stock solutions were dissolved in Milli-Q water. Tetracycline was purchased from Sigma and dissolved in dimethyl sulfoxide. Teicoplanin was purchased from Hoechst Marion Roussel and dissolved in dimethyl sulfoxide. All reagents were diluted in saline before use.

### Infection Experiments

**Figure 1:**
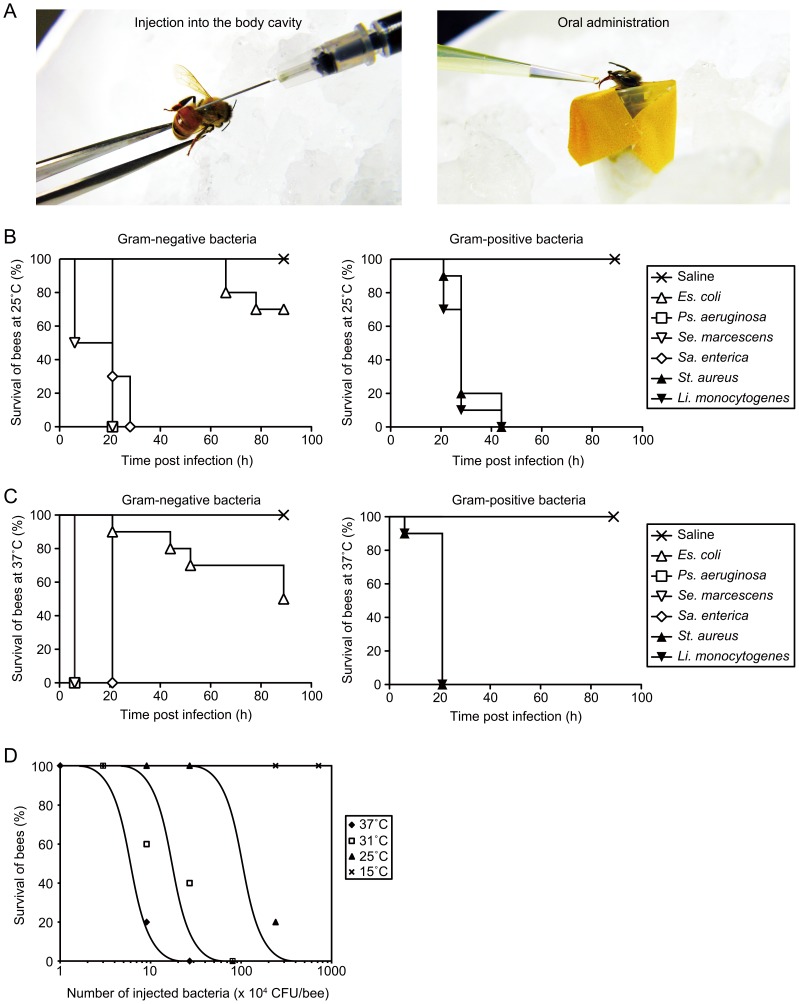
Establishment of a bacterial infection model in the honeybee. A, Images of sample injection into the body cavities of bees (left) and oral administration (right). In the left panel, bees anesthetized on ice were injected into the abdomen with 25 µl of each sample using a 1-ml syringe. In the right panel, anesthetized bees were placed in 1.5-ml tubes and fixed with paper tape. Ten microliters of sample containing sucrose was directly administered to bees extending the proboscis. B–C, Survival of in-hive worker bees (n = 20) after infection with Gram-negative (left) and Gram-positive (right) bacteria. Overnight culture of each bacterium was diluted 100-fold with saline. Survival numbers of bees were monitored at 25°C (B) or 37°C (C). The data shown are representative of three experiments with similar results. D, Effect of incubation temperature on survival of in-hive worker bees after *S. aureus* infection. LD_50_ values were determined 24 h after injection with a live bacterial suspension of *S. aureus* (n = 5).

In time-course assays, overnight bacterial cultures were diluted with saline by 100- or 200-fold, and the OD_600_ value was adjusted to 0.05∼0.10. Two-fold serial dilutions of bacterial cultures were prepared to determine the LD_50_ values. For injection experiments, bees anesthetized on ice were injected with 25 µl of each sample into the abdomen using a 1-ml syringe equipped with a 27-gauge needle (Terumo). For oral administration, bees anesthetized on ice were placed in 1.5-ml tubes and fixed with paper tape (Fig. 1A). Bees were recovered from anesthesia at room temperature and fed 10 µl of each sample containing 1 mol l^−1^ sucrose and antibiotics at various concentrations using a micropipette (Fig. 1A; [26]). Treated bees were maintained in a plastic dish and fed with 1.4 mol l^−1^ sucrose-soaked cotton at either 15°C, 25°C, 31°C, or 37°C. Infected honeybees were judged to be dead if no movement was observed in any parts of the body, antennae, or legs when touched by pipette tips. The LD_50_ values of bees at 24 h after infection were determined using Reed-Muench method, as previously described [8]. Survival data were plotted using Prism 5 (GraphPad Software, Inc.), and statistically significant differences between the survival curves were analyzed by the log-rank test.

## Results

### Killing of Honeybees by Injection of Human Pathogenic Bacteria

We first examined whether various bacteria known to be pathogenic against humans kill honeybees. Injection of the bacteria into the hemocoel of bees seems to mimic the pathology of sepsis in humans. Each suspension of Gram-negative (*Pseudomonas aeruginosa*, *Serratia marcescens*, and *Salmonella enterica*) and Gram-positive bacteria (*Staphylococcus aureus* and *Listeria monocytogenes*) was injected into the hemocoel of nurse bees anesthetized on ice (Fig. 1A). After the bees were transferred and maintained in plastic cages at either 25°C or 37°C, survival of the bees was monitored. Bees infected with either of the five pathogenic bacteria were killed within 2 days (Fig. 1B, C). In contrast, it took more than 3 days to kill 50% of bees infected with an avirulent laboratory strain of *E. coli* (KP7600) (Fig. 1B, C). Moreover, the amount of incubation time required for killing bees by infection with either of the five pathogens was lower at 37°C than at 25°C (Fig. 1B, C). We further examined the effect of temperature on killing of bees by *S. aureus* infection at 15°C, 25°C, 31°C, and 37°C. The LD_50_ value of *S. aureus* at 25°C was 5×10^5^ colony forming unit (CFU)/bee, whereas that at 31°C and 37°C was 2×10^5^ CFU/bee and 5×10^4^ CFU/bee, respectively (Fig. 1C). Bees incubated at 15°C were not killed even when infected with 8×10^6^ CFU/bee of *S. aureus* (Fig. 1C). Based on these results, we concluded that the LD_50_ values of *S. aureus* against honeybees decreased in response to an increase in temperature.

### Assessment of Pathogenesis of *S. aureus* Mutant Strains of Virulence Genes Using the Honeybee Infection Model

**Figure 2:**
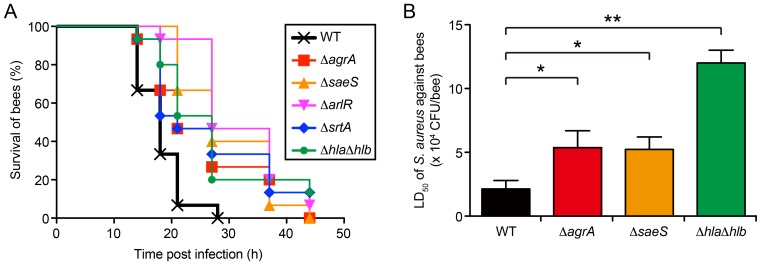
Killing of honeybees by *S. aureus* gene-disrupted mutants of virulence genes. A, Time-course of honeybee killing by *S. aureus* gene-disrupted mutants. Overnight culture of *S. aureus* NCTC8325-4 strain (WT; wild-type) or mutants (Δ*agrA*, Δ*saeS*, Δ*arlR,* Δ*srtA*, and Δ*hla*Δ*hlb*) was diluted 200-fold with saline. Survival of in-hive worker bees (n = 15) after infection with *S. aureus* mutants was monitored at 25°C. Statistical differences between the wild-type and each mutant were determined to be significant (p<0.05) by log-rank test. B, Determination of LD_50_ values of *S. aureus* mutants against honeybees. Survival of in-hive worker bees (n = 15) after 24 h of infection with *S. aureus* wild-type (WT) strain or mutants (Δ*agrA*, Δ*saeS*, and Δ*hla*Δ*hlb*) was monitored at 25°C, and the LD_50_ values were calculated. Data represent mean ± SDs of three experiments. Statistical analysis was performed by one-way ANOVA, and differences compared with the wild-type (WT) strain-infected group were analyzed by Dunnett’s multiple comparison tests (*, p<0.05; **, p<0.005).

We tested the killing ability of *S. aureus* gene-disrupted mutants of several virulence genes in the honeybee infection model. We focused on major virulence factors required for pathogenesis in mammals; the two-component systems that globally regulate virulence gene expression (Agr [11], [12], Sae [13], and Arl [10], [14]), the cell-surface modifier sortase A (encoded by the *srtA* gene) [27], and extracellular hemolysins (Hla and Hlb) [12]. *S. aureus* mutants of the *agrA*, *saeS*, *arlR*, *srtA* genes, and a double mutant of the hemoylsin genes (Δ*hla*Δ*hlb*) showed delayed killing of honeybees compared with the parent strain (Fig. 2A). Moreover, we demonstrated that LD_50_ values of Δ*agrA*, Δ*saeS*, and Δ*hla*Δ*hlb* strains increased by 3- to 6-fold (Fig. 2B). These results suggest that the honeybee model is suitable for assessing the contribution of virulence genes in bacterial pathogenesis.

### Bacterial Killing of Honeybees According to Division of Labor

**Figure 3:**
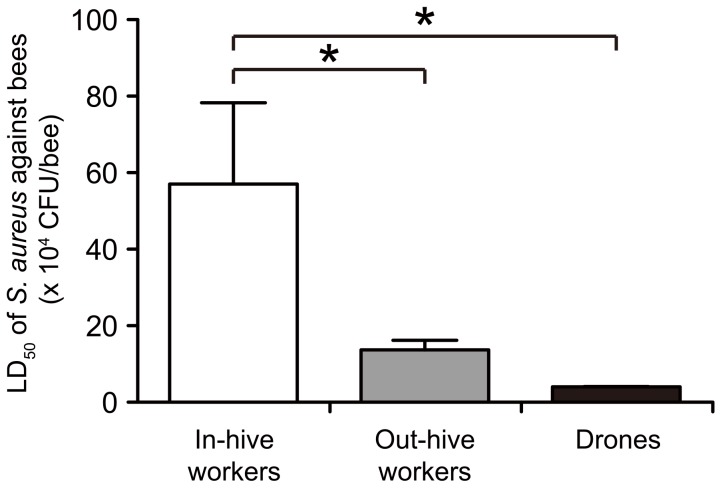
Comparison of susceptibilities to bacterial infection between honeybees in different labor divisions. LD_50_ values of *S. aureus* were determined 24 h after infection in either in-hive workers, out-hive workers, or drones (n = 5–8). Data represent mean ± SDs of three experiments. Statistical analysis was performed by one-way ANOVA, and differences compared with the in-hive workers group were analyzed by Dunnett’s multiple comparison tests (*, p<0.05).

Honeybee colonies comprise female workers engaged in different labors such as nurse bees, foragers, queens, and drones [22]. The variations in labors may lead to the emergence of differences in pathogen types that bees contact, as well as the immune reactiveness of infected bees. We tested the difference in host resistance against bacterial infections among the division of labors. The LD_50_ values of *S. aureus* were determined 24 h after injection into the hemocoel of either nurse bees, foragers, or drones. The LD_50_ value was much higher in nurse bees than in foragers and drones (Fig. 3). The high resistance to pathogens observed in nurse bees is reasonable for avoiding group infection within the nests. On the other hand, worker bees foraging outside the hives exhibit attenuated host resistance, possibly due to senescence (*i.e.,* old age) and alterations in their physiologic state.

### Therapeutic Effects of Antibiotics against Bacterial Infection in the Honeybee

**Figure 4:**
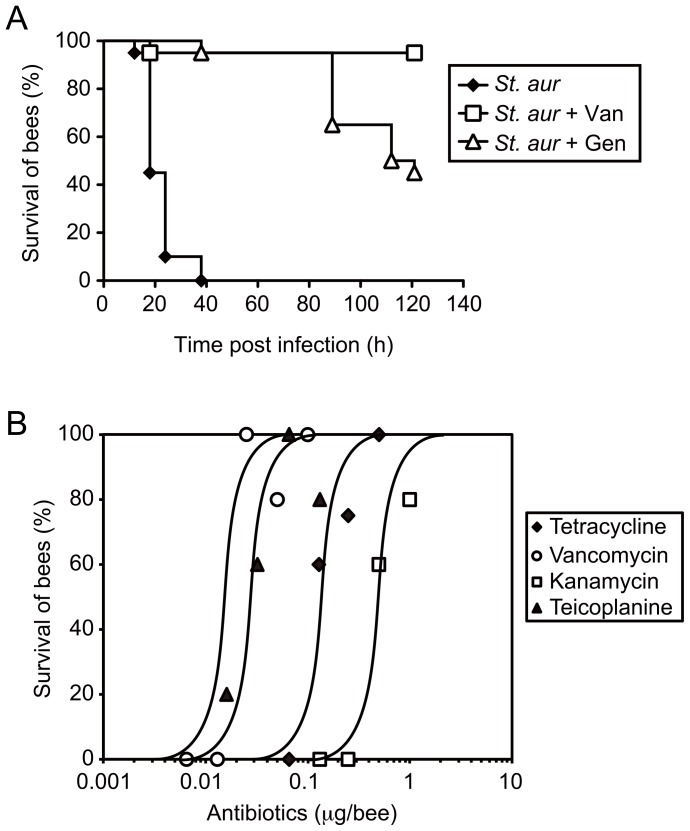
Therapeutic effects of antibiotics against bacterial infection in honeybees. A, Effect of co-injection of vancomycin or gentamycin on survival of in-hive worker bees infected with *S. aureus* (n = 20). B, Dose-dependency of therapeutic effects of antibiotics on *S. aureus* infection in in-hive worker bees. Survival numbers were determined 24 h after infection (n = 5).

We next tested if the above infection model using the honeybee as a host animal would be useful for *in vivo* evaluation of the therapeutic effects of antibiotics against pathogens. While more than half of the in-hive worker bees infected with *S. aureus* died within 24 h, bees co-injected with *S. aureus* and clinically effective antibiotics such as vancomycin or gentamycin lived longer than the control group (Fig. 4A). Various antibiotics, such as tetracycline, vancomycin, kanamycin, and teicoplanin showed therapeutic effects against *S. aureus* infection in honeybees in a dose-dependent manner (Fig. 4B), and the ED_50_ values were determined (Table 1). We previously examined the therapeutic effects of sev show effects by oral administration and injection ineral antibiotics in a silkworm infection model and demonstrated that the pharmacokinetics parameters of the drugs in silkworms were similar to those reported in mammals [16], [17]. The above ED_50_ values determined in the honeybee infection model were comparable with those reported in other models using silkworms and mice (Table 1, [16]). We then examined the effects of antibiotics administered orally in the honeybee-*S. aureus* infection model. Antibiotics with large molecular masses, such as vancomycin and kanamycin, are poorly absorbed from the intestines and therefore do not show therapeutic effects by oral treatment in mammals. We previously reported that these antibiotics failed toto the midgut of silkworms [16]. Here we demonstrated that tetracycline, which is efficiently absorbed from the digestive tract in silkworms and mammals [16], delayed the killing of bees by oral administration, whereas vancomycin and kanamycin failed to show therapeutic, effects even at doses as high as 330 µg/insect (Table 1). This finding is in good agreement with previous reports of the relationship between the enteric absorption and effectiveness of antibiotics in other model animals. Thus, we concluded that the honeybee infection model was suitable for *in vivo* assessment of therapeutic effects of antibiotics reflecting the pharmacokinetic aspects.

**Table 1:** ED_50_ values of antibiotics in the honeybee *S. aureus* infection model.

Antibiotics	*A. mellifera* L.	*A. mellifera* L.	*B. mori* a	*B. mori* a
	i.c.	p.o.	i.h.	p.o.
**Tetracycline**	0.2^b^	2	0.4	8
**Vancomycin**	0.02	>330	0.3	>400
**Kanamycin**	0.4	>330	3	>500

ED_50_ values of tetracycline, vancomycin, and kanamycin against *S. aureus* infection in in-hive worker bees (n = 5) were determined 24 h after infection. Antibiotics were injected into the cavities (i.c.) or orally administered (p.o.), and live bacterial suspension of *S. aureus* was injected immediately after antibiotic treatment.

a ED_50_ values of *B. mori* were previously reported [16].

b µg/insect.

## Discussion

Temperature is a critical factor that affects both the infection processes of pathogens and the physiologic state of the infected hosts. In *D. melanogaster*, the mortality of flies infected with *P. aeruginosa* and *Lactococcus lactis* is higher at 29°C than at 17°C [28]. In addition, the mRNA levels of antimicrobial peptides (AMP) in infected flies are higher at a low temperature, supporting the findings from infection experiments [28]. In the present study, we found that the susceptibility of honeybees against *S. aureus* was increased by an upshift in the incubation temperature. Under high temperature conditions, the effects of both the promotion of bacterial growth and the suppression of innate immune responses in bees seem to result in attenuated host resistance against bacterial infections, consistent with previous reports using other insect models such as *D. melanogaster* [28].

In our experimental conditions, host resistance against *S. aureus* infection was higher in nurse bees than in foragers and drones. The labors of worker honeybees change from nursing to foraging, dependent on their age [22]. Recently, Laughton *et al*. [29] reported that lipopolysaccharide challenge-dependent AMP production levels in old-aged foragers were lower than those in young bees. They also demonstrated that the AMP levels were lower in drones than in foragers at any age [29]. Moreover, both the induction levels of melanization, another humoral immune response in insects, and the ability of hemocytes to encapsulate foreign substances significantly decrease with age in worker bumblebees [30]. Although the direct cause of immunosenescence in old-aged foragers and drones was not determined, the authors of the above reports speculated that a trade-off between immune responses and energy costs for foraging behaviors and sperm production might explain the differences. Based on both our results and previous reports, we considered that an attenuated ability to induce innate immune responses such as AMP production and encapsulation after infection in old-aged foragers and drones lowers the resistance to pathogenic bacteria.

Recent reports demonstrated that honeybees possess immune systems, such as humoral and cellular immunity, similar to those found in other insects [24], [29], [31]–[33]. Basic reactions and pathways of innate immunity are conserved among species, whereas some of the repertoires differ between honeybees and other insects [24], [34]. Under our experimental conditions, *S. aureus* mutants of the two-component system regulators (*agr*, *sae*, and *arl*), sortase A (*srtA*) which is required for cell wall-protein anchoring, and hemolysins (*hla* and *hlb*) showed attenuated killing activity against honeybees. The critical roles of these genes for *S. aureus* pathogenesis are reported in various mammalian infection models [10]–[14], [27]. In addition, the findings in honeybee are consistent with those in other invertebrate infection models; the two-component system regulators Agr and Sae are required for virulence in both silkworm [15] and *Caenorhabditis elegans* [35], [36], and Hla contributes to host-killing in *C. elegans* [35], [36]. Our results showing consistency between honeybees and other models regarding the susceptibility to pathogens and the therapeutic effects of antibiotics seem to reflect the similarity in innate immunity among insects.

Social animals, including humans, are able to suppress the spread of pathogens among group members via social behaviors. In rats, for example, individuals whose immune systems are activated by microbial stimulants tend to be isolated or attacked by other healthy members within the group [37], [38]. Honeybee nestmates display aggressive behaviors toward infected individuals to eliminate them from the colony [39]. These behavioral alterations are thought to be induced by signals transmitted from infected individuals to the other members; the underlying mechanisms, however, have yet to be clarified. In the present study, we examined the susceptibility of isolated individual bees, but further studies are needed to examine the alterations in immune responses and hygiene behaviors in grouped colonies. To date, studies of infectious diseases using honeybees have focused mainly on diseases caused by pathogens that naturally infect bees, such as *Paenibacillus larvae* and *Nosema* [40]–[42]. In the present study, we established a quantitative model in honeybees for assessing bacterial infections and the therapeutic effects of antibiotics. In addition to their advantageous features as an infection model similar to other non-social insects, honeybees may provide a useful model in future studies of the relationship between infection and social behaviors.
